# Editorial: Multiscale Modeling of Rhythm, Pattern and Information Generation: from Genome to Physiome

**DOI:** 10.3389/fphys.2020.00281

**Published:** 2020-04-07

**Authors:** Shangbin Chen, Alexey Zaikin

**Affiliations:** ^1^Britton Chance Center for Biomedical Photonics, Wuhan National Laboratory for Optoelectronics-Huazhong University of Science and Technology, Wuhan, China; ^2^Department of Mathematics and Institute for Women's Health, University College London, London, United Kingdom; ^3^Lobachevsky State University of Nizhny Novgorod, Nizhny Novgorod, Russia; ^4^Centre of Complex Systems Analysis, Sechenov University, Moscow, Russia

**Keywords:** quantitative physiology, multiscale model, computational model, physiome, systems biology, complexity

The rapid advances of technology have revolutionized the research of physiology in the era of big data. More and more anatomical, physiological, and clinical data are collected to support precision medicine. Effective progress in research can be obtained only by merging data mining with modeling and analysis in the frame of *Quantitative Physiology*. *Quantitative Physiology* is the quantitative description, modeling and computational study of physiology, which is an increasingly important branch of systems biology. It will take the power of physics, mathematics, information technology, etc., to implement quantitative, testable, and predictable research to boost understanding the function in living systems. It is quite similar to mathematical physiology and computational physiology that address the theoretical or computational nature of physiology, but it is somehow different to stress data acquisition and quantitative description in the era of big data.

In 2003, the completion of the Human Genome Project (HGP) was announced effectively with the publication of the first complete human genome sequence. Due to the tight correlation between genes and functions, physiological genomics and functional genomics are hot topics in modern physiology. Among the long list of “ome” and “omics,” we should pay more attention to *Physiome* and *Physiomics*. *Physiome* comes from “physio-” (nature) and “-ome” (as a whole). The *Physiome* is the quantitative and integrated description of the physiological dynamics and functional behavior of the physiological (normal) and pathophysiological states of an individual or species (Bassingthwaighte, 2000). The *Physiome* describes the physiological dynamics of the normal intact organism and is built upon information and structure (genome, proteome, and morphome). Obviously, *Quantitative Physiology* matches very well with the definition of *Physiome*.

Stephen Hawking said that the next twenty-first century would be the century of complexity and indeed now Systems Biology or Medicine means dealing with complexity. This reality is that a huge amount of biological or physiological data, emerging from different-omics sources on very different scales, from genome to *Physiome*, exceeds our abilities to analyze these data. To link molecular and cellular events with physiological function and behavior is associated with wide ranges of space and time scales (Hunter and Borg, 2003). This Research Topic aims to provide state-of-the-art review of multi-scale modeling and data analysis to investigate the function in living systems, organisms, organ systems, organs, cells, and biomolecules carrying out the chemical or physical functions that exist in a living system. So far, this Research Topic has collected 14 articles (12 original researches and 2 mini reviews) that represent a cross sectional sample of *Quantitative Physiology*.

There are 4 typical modeling studies. The multi-scale cardiac computational modeling (Bai et al.) investigated the potential effects of a R858H mutation on the intracellular calcium handling, action potential profiles, action potential duration restitution curves, dispersion of repolarization, QT interval and spiral wave dynamics. It revealed that the L-type calcium current altered by mutation increases arrhythmia risk due to after depolarizations and increased tissue vulnerability to unidirectional conduction block. It provided a causal link between a R858H mutation and ventricular fibrillation. A neuron-astrocyte network model composed of 100 excitatory neurons and two astrocytes was developed (Gordleeva et al.). It showed that spatiotemporal properties of Ca^2+^ dynamics in spatially extended astrocyte can coordinate (e.g., synchronize) networks of neurons and synapses. In addition, A stochastic model of immune response (Fatehi et al.) and a simple spin glass-like model for the collective sensing of β-cells were proposed (Korosak and Rupnik).

Particularly, there are 4 papers of computational studies on deep brain stimulation (DBS). Tass' group reviewed that the dendritic and axonal propagation delays may lead to the emergence of neuronal activity and synaptic connectivity patterns, which cannot be captured by classic spike-timing-dependent plasticity models (Asl et al.). A short-term dosage regimen of coordinated reset stimulation could induce long-lasting desynchronization of the networks (Manos et al.). A novel stimulation method of pulsatile multisite linear delayed feedback was employed to modulate the pulse amplitude of high-frequency DBS (Popovych and Tass). And, a sham stimulation protocol for multichannel desynchronizing stimulation was proposed to provide controls (Zeitler and Tass). All these findings and implementations are helpful to develop therapeutic DBS.

The other 6 papers are all related with data analysis. Both short-time Fourier transform and multifractal detrended fluctuation analysis were used to seek the age-related signature of local field potential recordings (Makra et al.). Another work implemented the intelligent assessment and classification of ECG (Zhao and Zhang). Cross-correlation analysis were used in study of primary immunodeficiency diseases (Korsunskiy et al.) and development of brain neural networks (Mishchenko et al.). Both differential expression and network analysis were introduced in brain transcriptome (Wang and Wang) and functional genomics (Zhu et al.).

The work in this Research Topic involved the different levels of biomolecules, cells, organs, and different events of genetic expression, transcriptional regulation, calcium signaling, cell signaling, heart beating, brain integration. Here, a central concept is generation and processing of information. This Research Topic is devoted to set a paradigm for *Quantitative Physiology* by integrating biology, mathematics, physics or informatics. Both Editors of this Research Topic have joined the project “Digital Personalized Medicine of Healthy Aging (DPM-AGING): network analysis of Big Multi-omics data to search for new diagnostic, prognostic, and therapeutic targets.” This Research Topic is also a good example of *Quantitative Physiology*.

It is not easy to perform multi-scale modeling and data analysis to investigate the functions in living systems. Just as Schrödinger's question and answer: “How can the events in space and time which take place within the spatial boundary of a living organism be accounted for by physics and chemistry? The obvious inability of present-day physics and chemistry to account for such events is no reason at all for doubting that they can be accounted for by those sciences.”

## Author Contributions

All authors listed have made a substantial, direct and intellectual contribution to the work, and approved it for publication.

### Conflict of Interest

The authors declare that the research was conducted in the absence of any commercial or financial relationships that could be construed as a potential conflict of interest.
